# Early Preventive Intervention for Young Children With Sex Chromosome Trisomies (XXX, XXY, XYY): Supporting Social Cognitive Development Using a Neurocognitive Training Program Targeting Facial Emotion Understanding

**DOI:** 10.3389/fpsyt.2022.807793

**Published:** 2022-02-25

**Authors:** Nienke Bouw, Hanna Swaab, Sophie van Rijn

**Affiliations:** ^1^Clinical Neurodevelopmental Sciences, Department of Education and Child Studies, Leiden University, Leiden, Netherlands; ^2^Leiden Institute for Brain and Cognition, Leiden, Netherlands

**Keywords:** neurocognitive training, facial emotion understanding, sex chromosome trisomies, Klinefelter syndrome, triple X

## Abstract

**Background:**

Sex Chromosome Trisomies (SCTs; XXX, XXY, XYY) are genetic conditions that are associated with increased risk for neurodevelopmental problems and psychopathology. There is a great need for early preventive intervention programs to optimize outcome, especially considering the increase in prenatal diagnoses due to recent advances in non-invasive prenatal screening. This study is the first to evaluate efficacy of a neurocognitive training in children with SCT. As social behavioral problems have been identified as among the key areas of vulnerability, it was targeted at improving a core aspect of social cognition, the understanding of social cues from facial expressions.

**Methods:**

Participants were 24 children with SCT and 18 typically developing children, aged 4–8 years old. Children with SCT were assigned to a training (*n* = 13) or waiting list (no-training) group (*n* = 11). Children in the training group completed a neurocognitive training program (The Transporters), aimed to increase understanding of facial emotions. Participants were tested before and after the training on facial emotion recognition and Theory of Mind abilities (NEPSY-II), and on social orienting (eyetracking paradigm). The SCT no-training group and typically developing control group were also assessed twice with the same time interval without any training. Feasibility of the training was evaluated with the Social Validity Questionnaire filled out by the parents and by children's ratings on a Visual Analog Scale.

**Results:**

The SCT training group improved significantly more than the SCT no-training and TD no-training group on facial emotion recognition (large effect size; $\eta_{\text{p}}^{2}$ = 0.28), performing comparable to typical controls after completing the training program. There were no training effects on ToM abilities and social orienting. Both children and parents expressed satisfaction with the feasibility of the training.

**Conclusions:**

The significant improvement in facial emotion recognition, with large effect sizes, suggests that there are opportunities for positively supporting the development of social cognition in children with an extra X- or Y-chromosome, already at a very young age. This evidence based support is of great importance given the need for preventive and early training programs in children with SCT, aimed to minimize neurodevelopmental impact.

## Introduction

Between 1:650 and 1:1000 children are born with a Sex Chromosome Trisomy [SCT; (1)]. SCT is characterized by an extra X- or Y-chromosome compared to the typical karyotype of 46, XX in girls and 46, XY in boys. Intellectual functioning is typically within normal limits, although somewhat lower on average, and SCT is related to a profile of specific cognitive vulnerabilities, for example in areas of executive functioning, language and social cognition [see for reviews: (2, 3)]. Children and adolescents with SCT also show higher percentages of clinical diagnoses of neurodevelopmental disorders, such as Attention Deficit/Hyperactivity Disorder (ADHD) and Autism Spectrum Disorder [ASD; (4–6)].

As SCT is a condition that are associated with increased risk for neurocognitive vulnerabilities and related neurobehavioral problems, these genetic conditions may serve as naturalistic “at risk” models of neurodevelopment. More specifically, the presence of an additional X- or Y-chromosome is known to convergently impact the maturation of brain functions and networks involved in social adaptive cognitive and behavioral development, often referred to as the “social brain” (7, 8). Therefore, the use of specific genetic conditions as models of more common behavioral and cognitive developmental disorders can reveal insights into early neurodevelopmental pathways that contribute to neurodevelopmental and -behavioral dysfunction in children. Therefore, research of the impact of genetic conditions such as SCT on development and potential effective interventions supporting development will help to elucidate the linkages among genetic, neurocognitive and neurobehavioral development.

Due to recent advances in non-invasive prenatal testing technology [i.e., the introduction of the NIPT; (9, 10)], it is possible to identify SCT as early as prenatally, resulting in increasing diagnoses of SCT. Give this rise in prenatal diagnoses of SCT, there is not only the opportunity to prospectively investigate early development, but also the opportunity and urgent need to study whether early preventive interventions may possibly reduce risk for difficulties in adaptive functioning and psychopathology later in life (11). However, to date, there has been no research evaluating the potential effects of early and preventive neurocognitive training in SCT. The present study aims at providing in this.

In defining the targets for early intervention in SCT, a key area may present the social domain, considering that social adaptation is among the key domains of vulnerability in SCT (3, 6, 10). Underlying cognitive mechanisms that may drive the risk of these social behavioral difficulties are social cognitive functions, referring to the mental processes that are used to perceive and process social cues, stimuli and environments, and underpin social adaptive functioning (12). With respect to SCT, recent reviews identify social cognition as among the key areas of difficulty from school age on (3, 6). Although outcomes are variable, reported vulnerable social cognitive abilities include reading social signals from social gaze directions, facial emotion understanding, face processing (accuracy and reaction time) and Theory of Mind, referring to the attribution of mental states, intentions and emotions to others (13). Calculated effect sizes indicated high to very high clinical significance. Interestingly, specific age dynamics during early development of social cognitive functions in young children with SCT were recently found (14), often described as the “growing into deficit” phenomenon (15), the effect that development is increasingly deviating compared to typical developing peers when children become older. Early intervening with children who are “at risk” for adverse development, but do not yet exhibit full expression of the syndrome, may provide the best advantages from intervention. By implementing intervention early in life, the course of social development may be shaped, preventing for adverse long-term outcomes (16). Given the difficulties in underlying social cognitive mechanisms in SCT that serve as building blocks for social adaptive functioning (12), it is important to study whether it is possible to support the development of social neurocognitive functions early in development by early intervention trials.

Training of emotion perception and understanding appears to be an important component of effective social cognitive interventions (17). In addition, emotion recognition develops already early in life (18), is proven to be vulnerable across the life-span of individuals with SCT, and therefore an important target to preventively support social cognitive development early in life of individuals with SCT. In typical early social cognitive development, the ability to recognize facial expressions correctly and to respond to them appropriately is vital for successful everyday social interaction, and a prerequisite for showing social adaptive behavior, responsive of social feedback that follows social interactions. This ability to recognize facial emotions, in turn, depends on basic social orientation, the spontaneous visual orienting of attention to naturally occurring and meaningful aspects of social interactions (i.e., eyes and faces), which is already present in the first 6 weeks of postnatal life (19); for a review on eye tracking studies, see (20). Later developing and higher order social cognitive skills as for example Theory of Mind likely depend on this very early propensity to orient attention to social important information and to recognize facial emotions (21). Attributing and understanding mental states such as beliefs, desires and intentions of others and oneself with the ability to share these during social interactions (i.e., Theory of Mind) continue to develop throughout childhood, based on maturation of complex neural networks and high-order cognitive processes (22). We evaluated the efficacy of an emotion recognition training program in SCT on key areas of typically social cognitive development during early childhood that were found to be vulnerable in young children with SCT, i.e., on measures of emotion recognition, Theory of Mind and social orientation (14).

Most of the currently available training programs targeted to enhance emotion recognition are computer-based. These computer-based neurocognitive training programs provide the opportunity to teach emotion recognition in a controlled and structured environment with little social demands. Motivation and interest are usually easily to maintain, and materials are low-budget and therefore easy accessible (23, 24).

The efficacy of a home-based computer-based emotion recognition training in young children with SCT in the current study was evaluated by comparison of measures of emotion recognition, Theory of Mind and social orienting before and after the training. Training effects in young children with SCT were compared with two groups that did not attend the training program i.e., a waiting list group with SCT, and a group of typically developing children. Also, we studied feasibility and implementation of the training program in the SCT training group, based on self-report of the parents, and the children. Because studies on the efficacy of neurocognitive training that target to support early social cognitive development in SCT are lacking, this study is unique and may provide important implications for clinical care and future research aimed at improving evidence-based care for children with SCT in order to support optimal neurodevelopmental outcome.

## Materials and Methods

### Recruitment

The present study is part of a larger ongoing longitudinal study (the TRIXY Early Childhood Study–Leiden, The Netherlands), which includes children with SCT and nonclinical controls. The TRIXY Early Childhood Study aims to identify neurodevelopmental risk in young children with an extra X or Y chromosome. Recruitment and assessment of the current study took place as part of this larger study, at the Trisomy of the X and Y chromosomes (TRIXY) Expert Center at Leiden University (LUBEC) in Leiden, The Netherlands. Children in the SCT group were recruited in cooperation with clinical genetics departments in the Dutch speaking parts of Western Europe.

Typically developing control children were recruited from the western part of The Netherlands, and approached with information brochures about the study. All participants were Dutch speaking, had normal or corrected-to-normal vision, and did not have a history of traumatic brain injury. The diagnosis of SCT was defined by trisomy in at least 80% of the cells, which was confirmed by standard karyotyping. For ethical reasons, children in the typically developing group were not subjected to genetic screening. As the prevalence of SCT is ~1 in 1,000, the risk of having one or more children with SCT in the typically developing group was considered minimal and acceptable.

### Participants

A group of 25 children with SCT (range 4–8 years old) was included in this study. Children with SCT were assigned to a training group (SCT training group; *n* =14) or waiting list no-training group (SCT no-training group; *n* =11). See Table 1 for background information of the participants. Likelihood Ratio tests were performed to investigate the ratios of age, gender and karyotype distribution across the study groups. Mean age across the three study groups did not differ between the three groups [*F*_(2, 39)_ = 0.58, *p* = 0.566]. Also, gender distribution did not differ between the three study groups [λ(2) = 2.96, *p* = 0.227]. There was no difference in distribution of karyotypes between the SCT training and SCT no-training group [λ(2) = 3.50, *p* = 0.187]. One girl with 47, XXX in the SCT training group dropped out of the study during the second week, as she was not motivated to continue watching the training episodes any longer.

**Table 1:** Background information of participant.

	**SCT training group**	**SCT no-training group**	**TD control group**
Age	5.86 (1.28)	6.33 (1.27)	5.87 (1.15)
Gender	5 boys, 8 girls	8 boys, 3 girls	9 boys, 9 girls
Parental education	5.58 (1.08)	6.10 (0.83)	5.58 (1.41)
Karyotype	8 XXX, 4 XXY, 1 XYY	3 XXX, 5 XXY, 3 XYY	n.a.
Recruitment strategy^*^	A:6, B:4, C:3	A:8, B:1, C:2	n.a.

*SCT, Sex Chromosome Trisomy; TD, Typically Developing*.

* *A = Prospective follow-up, B = Information seeking parents, C = Clinically referred cases*.

For the SCT group, recruitment strategy was assessed, and three subgroups were identified: (1) “active prospective follow-up”, which included families who were actively followed after prenatal diagnosis (58.3% of the SCT group), (22) “Information seeking parents”, which included families who were actively looking for more information about SCT without having specific concerns about the behavior of their child (20.8% of the SCT group), and (3) “Clinically referred cases”, which included families seeking professional help based on specific concerns about their child's development (20.8% of the SCT group). The distribution of recruitment strategy did not differ between the SCT training and SCT no-training group [λ(2) = 2.25, *p* = 0.325]. One out of nine boys with 47, XXY had received testosterone treatment (11%). Testosterone treatment was performed at the age of 1 year, 3 years before the start of the current intervention study.

Parental education of the primary caregiver was assessed, according to the criteria of Hollingshead (25). Scores of this scale include: 0 (no formal education), one (less than seventh grade), two (junior high school), three (partial high school), four (high school graduate), five (partial college or specialized training), six (standard college/university graduation), and seven (graduate/professional training). Eighty-one percentage of all parents indicated that their child has a second caregiver. If two parents were available, level of education was averaged over both parents. No differences in parental education distribution between the three study groups were found [λ(16) = 15.36, *p* = 0.498].

### Design of the Study

**Figure 1:**
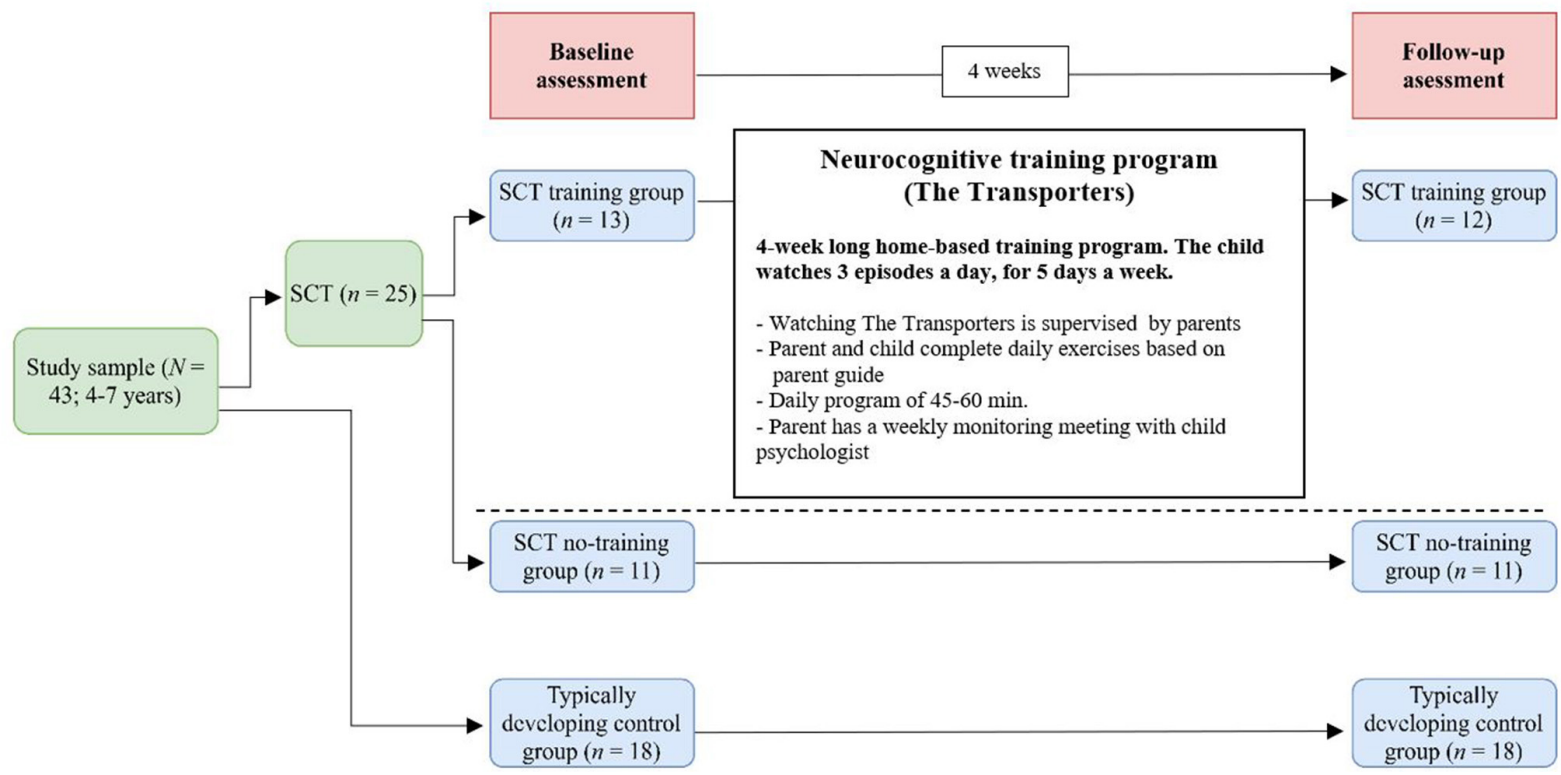
Flow diagram of the study design with the SCT training group, the SCT no-training group and a typically developing control group. SCT, Sex Chromosome Trisomie.

The current study had a repeated measures within-subject design. All participants were assessed twice: at baseline and 4 weeks later (follow-up). At baseline global level of cognitive functioning was measured, as well as receptive and expressive language skills. At both baseline and follow-up children facial emotion recognition, Theory of Mind, and social orienting was assessed. Between the baseline and follow-up assessment, children in the SCT training group participated in the emotion training program. Children in the SCT no-training group and the typical control group completed the baseline and follow-up assessment, but did not participate in the training program between baseline and follow-up, nor in other forms of early intervention as part of regular care. See Figure 1 for an overview of the study design.

### Emotion Training Program: The Transporters

**Figure 2:**
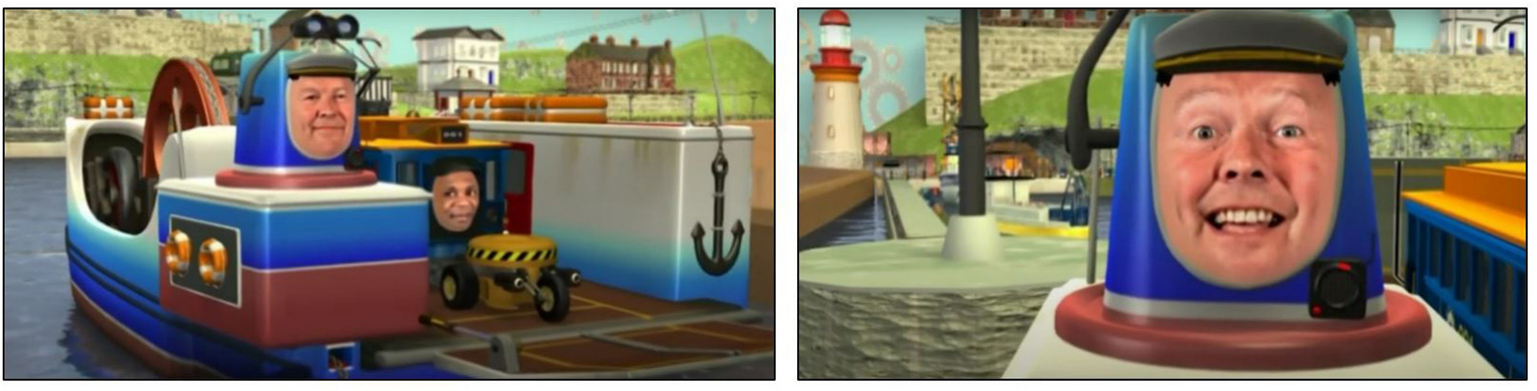
Screenshot of an episode from The Transporters training program: character William showing the emotion “happy”.

The Transporters is a narrated and animated DVD series and was originally developed to teach young children between the age of three and eight about emotions, their causes and consequences, and their corresponding facial expressions [Changing Media Development, www.thetransporters.com; (26)]. The series consists of fifteen 5-min episodes, portraying key emotions including basic emotions and nine more complex emotions: happy, sad, angry, afraid, disgusted, surprised, excited, tired, unfriendly, kind, sorry, proud, jealous, joking and ashamed. The narrated stories are built around eight characteristics who are vehicles (e.g., trams, cars, railway) with real-life faces of actors showing the emotions. The emotion is presented in the context of the series plot, in which the emotions are labeled, facial expressions are highlighted, and the context of the emotional experience is provided within social interactions between the toy vehicles. The assumption behind The Transporters is that through repetitive watching of entertaining episodes children might enhance facial emotion recognition and understanding skills [see for an extensive description of The Transporters: (27)]. The Transporters has been proven to be successful in improving emotion understanding abilities in young children with ASD (27, 28), although mixed results were found with respect to efficacy of The Transporters in young children with ASD with a lower range of cognitive ability (29, 30). See Figure 2 for screenshots of the first episode.

Children in the SCT training group watched the Dutch version of The Transporters in their home setting (De Ambelt, The Netherlands; resources.autismcentreofexcellence.org); they watched three episodes a day supervised by the parent, for 5 days a week and 4 weeks long (see Figure 1). The episodes were repeated in the same order every single week, in order to achieve repetitive watching of the episodes. Parents were provided with a detailed manual that consists of operating instructions, and a daily diary with a general introduction to the separate episodes, and exercises and questions to discuss with their child after watching the episodes. These exercises and questions were aimed to broaden the child's understanding of the emotional concepts as presented in the episodes, and to facilitate consolidation of learned skills. Examples of exercises and questions are: “Who is kind to you when you are in a bad mood? What does this person do? How does that make you feel? What do you do when you see that your mother/father/brother/sister/friend is sad or worried?” During the training period, the parent had a weekly call with the researcher to discuss and find solutions for any practical bottlenecks.

### Instruments

#### Background Measures: Global Level of Cognitive and Language Development

At baseline, global level of cognitive and language development was assessed in all children. Four subtest of the WPPSI were used to estimate global level of intelligence [Block Design, Matrix Reasoning, Vocabulary, and Similarities; (31)]. Total IQ estimates were calculated based on this short form version of the WPPSI-III (32). The Peabody Picture and Vocabulary Test [PPVT; (33)] was used to measure receptive language level. To measure expressive language skills, the Clinical Evaluation of Language Fundamentals-Preschool, 2^nd^ edition [CELF-Preschool; (34)] was administrated.

#### Facial Emotion Recognition

The Affect Recognition subtest of the NEPSY-II neuropsychological test battery (35) was used to assess children's ability to discriminate among common facial emotions from photographs of children, and was administrated at baseline and follow-up. The task has been normed with typically developing children aged 3–16 years old. During the task, participants are required to match faces of different children who show the same emotional expressions (happy, sad, angry, disgust, fear and neutral). The participant indicates if two expressions are the same or different, determines which two faces have similar expressions, or identifies two children with expressions that match a third child's face. The total raw score range is between 1 and 35, with higher scores reflecting a better ability to recognize facial expressions.

#### Theory of Mind

The ToM subtest of the NEPSY-II neuropsychological test battery (35) was used to assess children's understanding of mental states and other people's perspectives at baseline and post-training. The ToM subtest consists of two different subtasks: verbal tasks and contextual tasks. In the verbal tasks, the questions are based on verbal scenarios with (six items) or without (11 items) support of pictures. They measure the understanding of (false) beliefs, intentions, other's thoughts, ideas and comprehension of figurative language. Two items aim to measure the child's verbal and gestural imitation abilities, as imitation abilities are thought to be a basic ability for ToM skills. The child is asked to answer the tasks verbally, with the exception of an imitation question where the child is asked to imitate gestures or words. In all of the items the child can answer very briefly; one word is often sufficient for a correct answer, and in two of the questions it is also possible to answer by pointing. The contextual tasks of the ToM subtest aim to measure the child's ability to relate affects to a broader social context. In these items the child is shown drawings with children in social contexts. In each drawing there is a target girl whose face is not shown. The child is asked to select one of four photographs of the same girl's face with different emotions selecting the emotion of the girl in the drawing. The child can answer by pointing. The total score range is between 1 and 28 (sum score of the 15 verbal tasks and six contextual tasks), with higher scores reflecting better ToM skills.

#### Social Orienting

**Figure 3:**
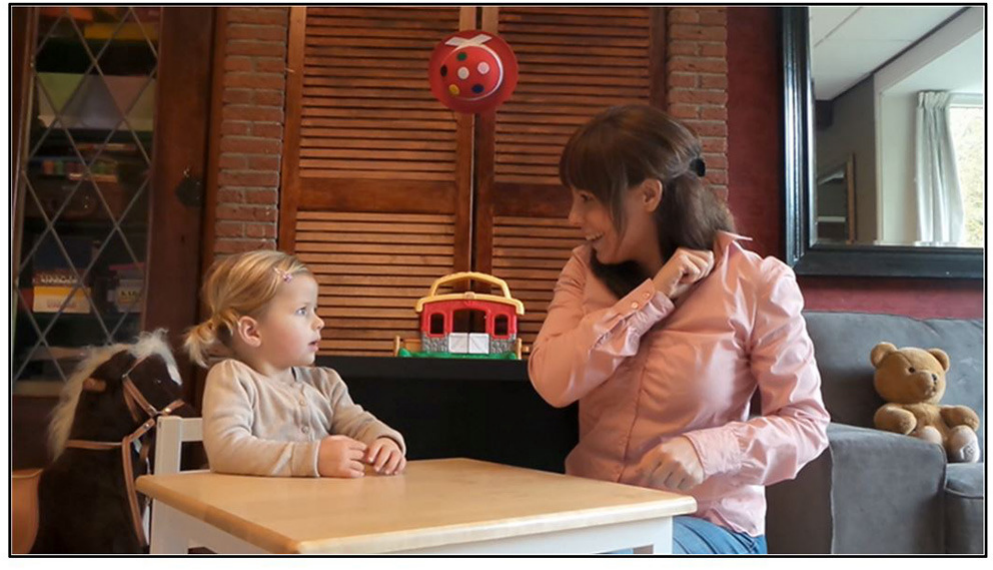
Screenshot of the video clip in the Social Orienting Paradigm.

Eye gaze fixations toward key sources of social information (eyes, faces) were measured during a Social Orienting Paradigm (see for a detailed description of the paradigm: 14), at both baseline and follow-up. The 30 s during video showed a social plot, in which social cues are reciprocally exchanged between a child and an adult. To prevent interference with language abilities, language used in the clip was not the same as the language of the participants (i.e., Italian vs. Dutch). In a group of non-clinical young children aged 3–7 years, this eyetracking paradigm was found to be related to real-life social behaviors, and independent of age, IQ, or gender (36). See Figure 3 for a screenshot of the video clip.

### Eyetracking Equipment and Procedures

Gaze data within specific areas of interest (AOIs) was collected using the Tobii X2-60 eyetracker (Tobii Technology AB, Danderyd, Sweden), which records the X and Y coordinates of the child's eye position at 60 Hz by using corneal reflection techniques. The computer with eyetracker was placed on a table adapted to the height of the seat, and the child was seated in a car seat at 65 cm viewing distance. A 5-point calibration procedure was used, with successful calibration defined as a maximum calibration error of one degree for individual calibration points (i.e., < 1 cm at a distance of 65 cm from the eyetracker). After the calibration procedure, the child was instructed to watch the movie clips and pictures on the computer. The paradigm started with an attention grabber (e.g., a moving picture of an animal, shown on a black background and accompanied by a sound) to direct the attention of the child to the screen.

Gaze data was processed using Tobii Studio (version 3.2.1), using the Tobii Identification by Velocity Threshold (I-VT) fixation filter. A fixation was registered if the velocity threshold for an eye movement exceeded 30°/s, and therefore controls for validity of the raw eyetracking data making sure only valid data were used (37). The “Dynamic AOI” tool was used to draw AOIs, drawn with a one centimeter margin, to ensure that the AOIs were sufficiently large outside the defining contours to reliably capture the gaze fixation (38). Dynamic AOIs were grouped into the following categories: eyes, faces and the whole screen. In order to evaluate the amount of nonvalid eye tracking data, the total visit duration toward the whole screen was calculated, divided by the duration of the clip, multiplied by 100, reflecting the percentage of valid data collected during each of the eye tracking tests. Proportions fixation duration were calculated by taking the total fixation duration within the AOI, divided by the total visit duration toward the whole screen of the individual child, multiplied by 100, reflecting the percentage of time children were attending to an AOI.

### Feasibility of the Emotion Training Program

#### Social Validity Parents

The Social Validity Questionnaire [SVQ; (39)] was filled in by the primary caregiver of the children in the SCT training group at follow-up, and assessed the parent's perception of: (a) how easy the training was to incorporate into daily life; (b) how easy the training was to learn; and (c) whether the training was effective for the child and family. The SVQ consist of 15 items, and were rated on a five point Likert scale ranging from one “Totally disagree” to five “Totally agree”. Examples of items are: “This training was easy to incorporate into my family life”, “This training was not complicated to learn” and “I noticed meaningful increases in my child's social interaction with the people in his environment”.

#### Perception of Children About the Training

**Figure 4:**
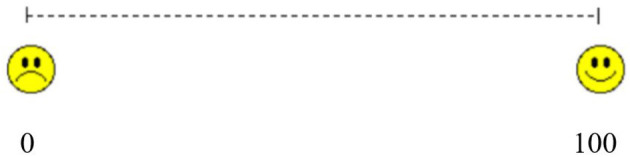
Visual Analog Scale used during the training program.

Children in the SCT training group reported how much they liked the training episodes in a daily diary. Their perception about the training was rated on a Visual Analog Scale (VAS) at a daily basis on an interval from 0 to 100 (see Figure 4). According to Shields et al. (40), only responses that were properly marked on the VAS lines (the mark must be a single vertical line that is no more than 1 mm away from the VAS line) and responses that were marked along the entire length of the VAS line (as opposed to using just the end-points and/or the middle of the VAS line) were used in the analysis. A total score was computed for each child by taking the sum of the registrations divided by the number of registrations (with a maximum of 20 registrations). Furthermore, how much children liked the training was calculated for every single week.

### Study Procedures

Assessments ate baseline and follow-up took place in a quiet room at the university or at home. Administration of the WPPSI, PPVT, CELF and NEPSY was performed on a table by trained child psychologists. The eyetracking procedure took place after the neurocognitive tests administration. The laptop with the eyetracker was placed in a small tent to standardize the testing environment, and to control for light conditions. The child was seated in a car seat in front of the eyetracker. The examiner was seated beside the child (directing Tobii Studio with a remote keyboard), and started the calibration procedure. Parents were allowed to stay in the room (out of sight) and were asked not to communicate with their child during the procedure. The Social Validity Questionnaire was filled in by the primary caregiver of the child.

### Ethical Approval and Informed Consent

This study was approved by the Ethical Committee of Leiden University Medical Center, The Netherlands. Signed informed consent was obtained from the parents of all participating children, according to the declaration of Helsinki.

### Data Analyses

Data were analyzed using the Statistical Package for the Social Sciences (SPSS), version 25. Baseline difference between study groups on background measures (global cognitive and language level) were analyzed with ANOVAs. In order to analyze the training effects, Repeated Measures MANOVAs were used with Time (baseline, follow-up) as within variable and Group (SCT training, SCT no-training, typically developing) as between variable. The interaction effect (Time x Group) was used to evaluate overall training effects. *Post-hoc* paired sample *t*-tests were carried out to analyze change in social cognitive abilities within the three study groups (SCT training, SCT no-training, typically developing). Repeated Measures MANCOVAs were carried out to test training effects, while covarying for cognitive and language abilities. Change in reported perception of children about the training was analyzed with a RM ANOVA. Level of significance was set at *p* < 0.05, two-tailed. Effect sizes were calculated with Cohen's *d* or partial η^2^ when applicable.

## Results

### Background Measures

Mean scores on cognitive background measures at baseline (global cognitive level, receptive and expressive verbal ability) are presented in Table 2. The three study groups do not differ on expressive verbal ability [*F*_(2, 39)_ = 2.35, *p* = 0.108]. However, the groups significantly differ in global cognitive functioning [*F*_(2, 39)_ = 7.64, *p* = 0.002], indicating lower functioning in the SCT groups (training and no-training), compared to the TD group. Both SCT groups perform similar on global cognitive functioning. Also, a significant difference on receptive verbal ability is found [*F*_(2, 39)_ = 5.39, *p* = 0.009], indicating lower ability in the SCT no-training group, compared to the TD group. No difference between the SCT training group and the no-training group was found for receptive verbal ability. Because of these differences between the SCT groups and the TD group, global cognitive functioning and receptive verbal ability are added as covariates in the analyses.

**Table 2:** Means (SD's) and group differences on cognitive and language functioning.

	**SCT training**	**SCT** **no-training**	**TD** **control**	***p-*value**	**Effect size ($\eta_{\text{p}}^{2}$)**	**Group differences**
	***n* = 13**	***n* = 11**	***n* = 18**			
**Global cognitive functioning** standard score; WPPSI-III	92.77 (10.68)	91.09 (14.98)	107.78 (13.20)	0.002	0.28	A, B < C
**Receptive verbal ability** standard score; PPVT-III	103.00 (11.10)	97.82 (12.33)	111.39 (10.55)	0.009	0.22	B < C
**Expressive verbal ability** scaled score; CELF	9.31 (2.66)	8.36 (2.87)	10.33 (1.85)	0.108	0.11	

*SCT, Sex Chromosome Trisomies; TD, typically developing; A, SCT training group; B, SCT no-training group; C, typical developing control group*.

### Training Effect: Facial Emotion Recognition

**Figure 5:**
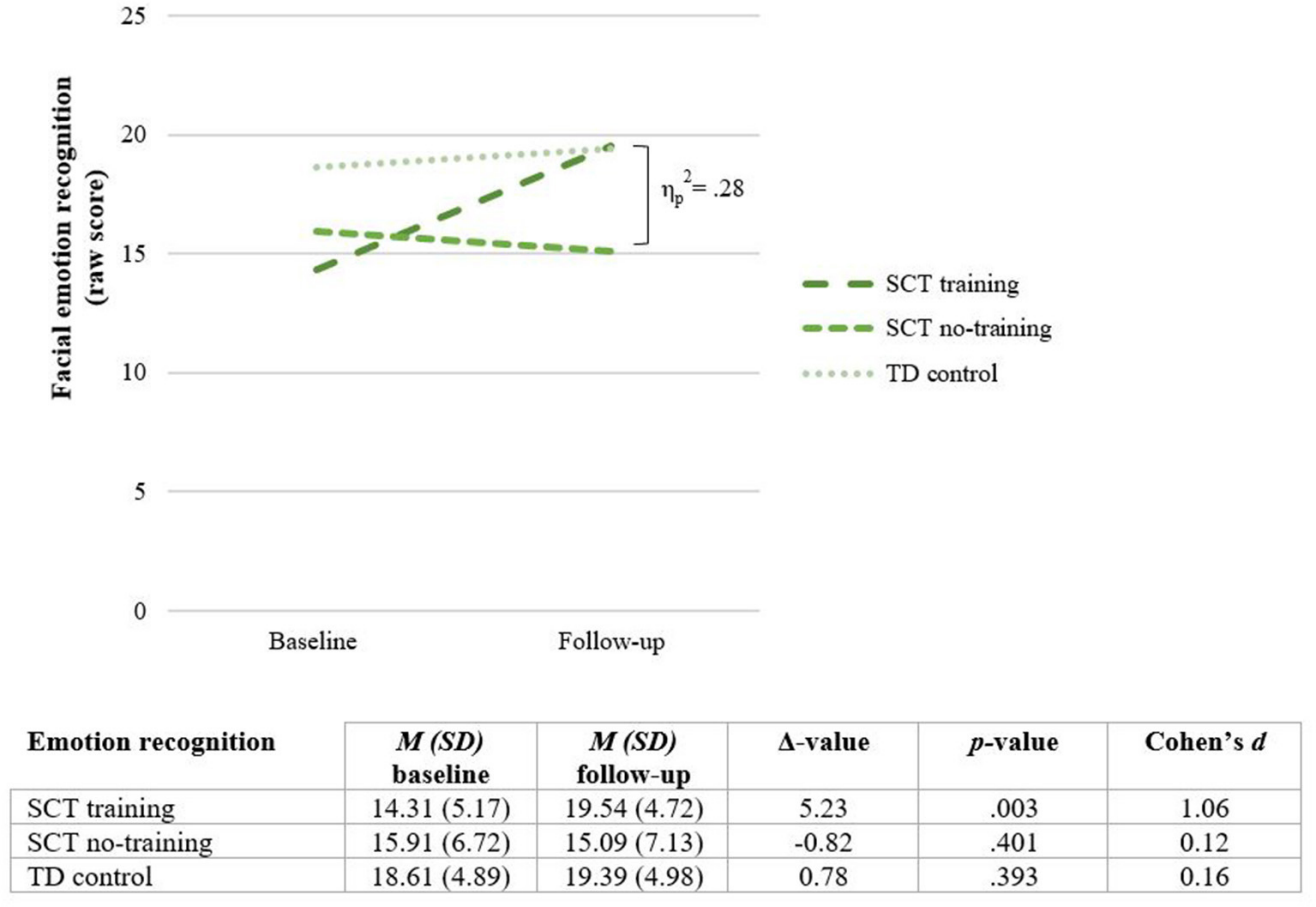
Training effect on facial emotion recognition: mean scores before (Baseline) and after completing the training (Follow-up). SCT, Sex Chromosome Trisomies; TD, typically developing.

First, to evaluate the overall effect of the training a RM MANOVA is conducted with Time (baseline, follow-up) as within variable and Group (SCT training, SCT no training, typically developing) as between variable. The analysis yield a significant interaction effect for Time x Group [*F*_(2, 39)_ = 7.50, *p* = 0.002, $\eta_{\text{p}}^{2}$ = 0.28), with a large effect size. This significant interaction effect on emotion recognition skills remains, even when global intelligence and receptive language skills are added as covariates [*F*_(2, 37)_ = 6.65, *p* = 0.003, $\eta_{\text{p}}^{2}$ = 0.26]. Next, *post-hoc* paired sample *t*-tests are used to analyze the effect of Time within the three study groups. In the SCT training group, a significant change in emotion recognition abilities is found [*t*_(12)_ = −3.72, *p* = 0.003]. In the SCT no-training group, no significant change in emotion recognition is found [*t*_(10)_ = 0.88, *p* = 0.401], neither in the typically developing group [*t*_(17)_ = −0.88, *p* = 0.393]. These findings indicate a significant change in emotion recognition abilities in the SCT training group, that is not present in the SCT no-training group or typically developing control group. After completing the training, the SCT training group (*M* = 19.54, *SD* = 4.72) scores comparable to their typically developing peers [*M* = 19.39, *SD* = 4.98; *t*_(29)_ = 0.84, *p* =0.933]. In terms of standard deviations, children in the SCT training group score 1.11 *SD* higher as compared to their average baseline score. See Figure 5 for an illustration of the interaction effect on facial emotion recognition.

### Training Effect: Theory of Mind

**Figure 6:**
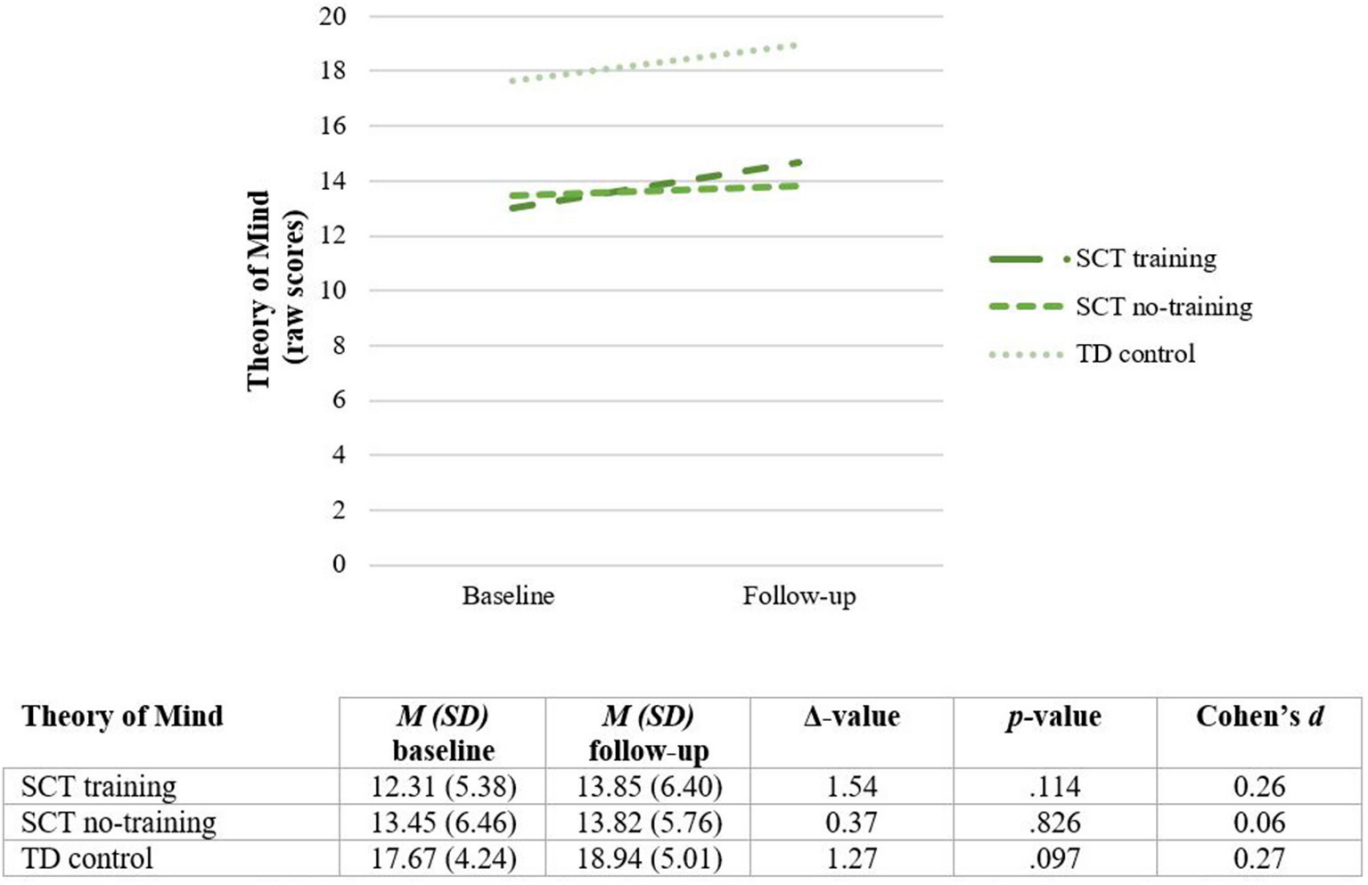
Theory of mind (total scores) before (Baseline) and after completing the training (Follow-up). SCT, Sex Chromosome Trisomie; TD, typically developing.

First, to evaluate the overall training effect on Theory of Mind, a RM MANOVA is conducted. No significant interaction effect is found for Time x Group **[***F*_(2, 39)_ = 0.31, *p* = 0.738] indicating no training effect on Theory of Mind. These findings do not change when global intelligence and receptive language skills are added as covariates [*F*_(2, 37)_ = 0.73, *p* = 0.488]. See Figure 6.

### Training Effect: Social Orienting to Eyes and Faces (Eyetracking Paradigm)

*Attention to the screen*. The Social Orienting Paradigm was successfully completed by 42 children at baseline, and 41 children at follow-up (one boy with 47, XXY in the SCT no-training group was not able to complete the task at follow-up). At baseline, the total proportion valid on-screen fixation duration is 95.5%, indicating sufficiently high attention to the screen. Attention to the screen do not significantly differ between the three study groups, [*F*_(2, 39)_ = 1.76, *p* = 0.186]. Similar at follow-up, the total proportion valid on-screen fixation duration is 94.3%, and do not significantly differ between the three study groups, [*F*_(2, 38)_ = 1.76, *p* = 0.186].

#### Training Effect

**Figure 7:**
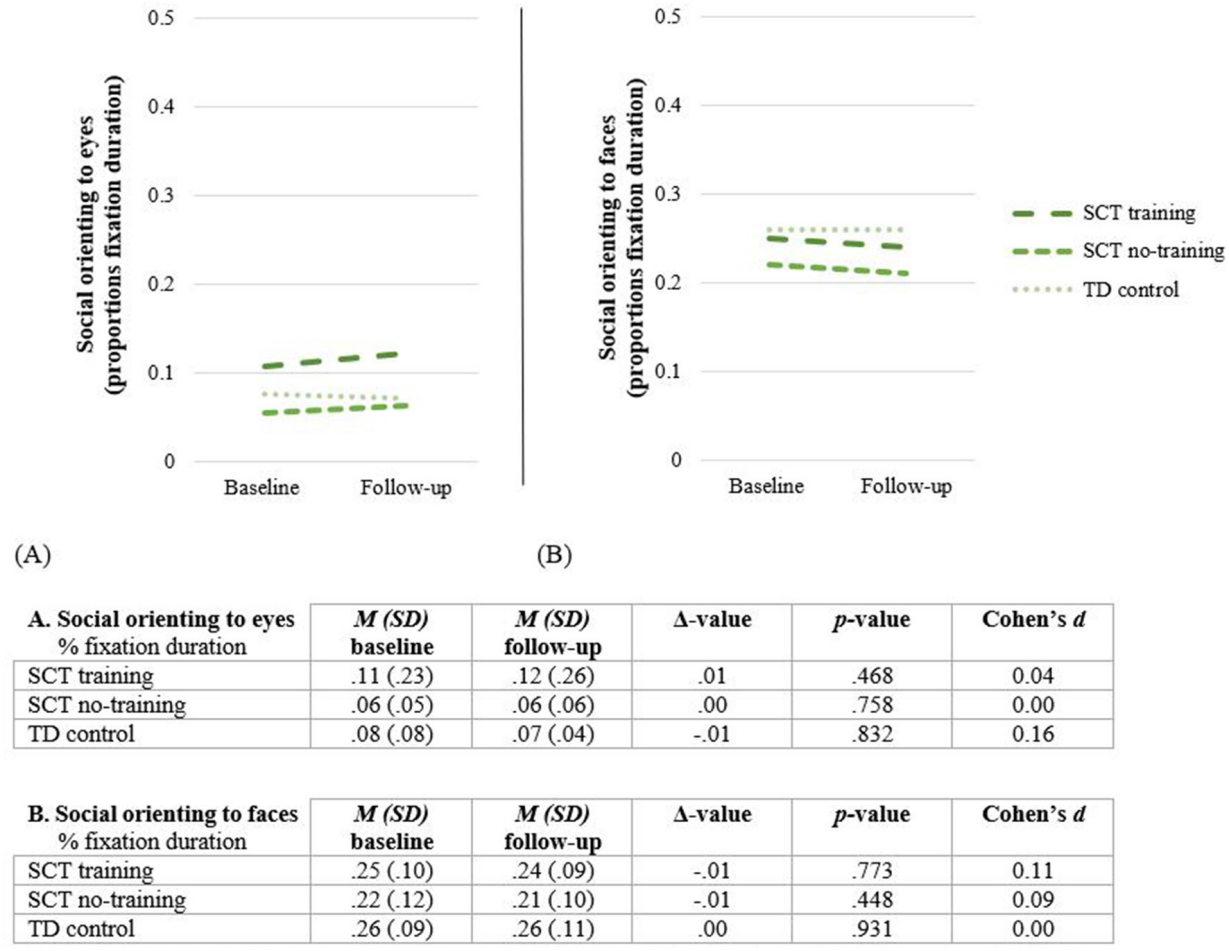
Social orienting to eyes **(A)** and faces **(B)** before (Baseline) and after completing the training (Follow-up). SCT, Sex Chromosome Trisomie; TD, typically developing.

A RM ANOVA is conducted to analyze overall training effect, revealing no significant effect of Time x Group for social orientation to eyes [*F*_(2, 38)_ = 0.22, *p* = 0.803] neither to faces [*F*_(2, 38)_ = 0.05, *p* = 0.948]. These findings do not change when global intelligence and receptive language skills are added as covariates [eyes: *F*_(2, 36)_ = 2.15, *p* = 0.131; faces: *F*_(2, 36)_ = 0.29, *p* = 0.741]. See Figure 7.

### Feasibility of the Emotion Training Program

#### Social Validity Parents

Response on the 5-point Likert scale of the 15 items of the Social Validity Questionnaire are recoded into three categories: negative opinion (value 1 and 2), a neutral opinion (value 3), and a positive opinion (value 4 and 5). Table 3 presents parents' responses on the three subscales of the SVQ. In sum, parents report positive experiences after the training period. To illustrate, 91.7% of the parents report that the training is easy to implement in daily life, 66.7% reported that the training is easy to learn and to use and valuable for their child. Although 83.3% of the parents recognize positive changes in their child after intervention (Q11: This intervention provided a significant positive change for my child), a vast majority of the parents are neutral about the generalization of the intervention to other contextual situations (Q12: I noticed meaningful increases in my child's interaction with the people in his environment; Q15: Other people noticed a significant positive change in my child). Only 8.3% of the parent report positive increases in child's eye contact (Q14: I noticed meaningful increases in my child's eye contact with the people in his/her environment). Parents do report that they would recommend the intervention to other parents and that they are willing to continue using the intervention model in the future. See the Supplementary Table 1) for parents' response on all items of the SVQ.

**Table 3:** Social validity of parents in the SCT training group on subscales of the Social Validity Questionnaire.

	**Negative opinion**	**Neutral opinion**	**Positive opinion**
	**Strongly disagree/disagree**	**Neutral**	**Agree / strongly agree**
Incorporation in daily life	8.3%	0%	91.7%
Easiness to learn the training	0%	33.3%	66.7%
Effectiveness of the training	16.7%	58.3%	25.0%

*N = 13*.

#### Perception of Children About the Training

**Figure 8:**
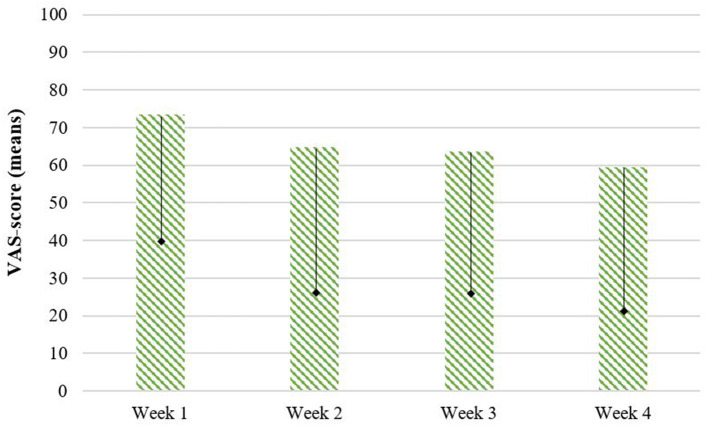
Perception about the training during the training program, reported by children on a Visual Analog Scale (VAS). = *SD* (only lower bar depicted).

Children were asked daily how much they liked the training, on a Visual Analog Scale. The mean VAS-score of children during watching the training episodes for the SCT training group is 65.2 on a scale of 0–100. There is no significant difference in VAS-scores of children reported between the first training week (*M* = 73.40, *SD*= 26.62), the second week (*M* = 64.76, *SD* = 30.31), the third week (*M* = 63.67, *SD* = 29.17), and the fourth week (*M* = 59.43, *SD* = 30.07), *F*_(3, 24)_ = 1.70, *p* = 0.194 (see Figure 8).

## Discussion

There is a great need for evidence-based interventions that support early development of young children with Sex Chromosome Trisomies (SCT). The current study aims to evaluate the effectiveness of a neurocognitive training in young children with SCT, aged 4–8 years. As social cognitive and behavioral vulnerabilities have been identified as among the key areas of vulnerability in SCT (6, 41), this neurocognitive training was targeted at improving the understanding of social cues from facial expressions.

Efficacy of the training was examined on key aspects of early social cognition that have proven to be vulnerable in young children with SCT: facial emotion recognition, Theory of Mind and social orienting (14). Three study groups were included in the study: 4–8 year old children with SCT, and two age- and gender-matched control groups that did not complete the training: one waiting list group with SCT and one typically developing group. Promising results regarding the effectiveness of the training were found, revealing that attending the 4-week home based neurocognitive training was effective in significantly improving the ability to identify and match basic and complex facial expressions in children with SCT, with a large effect size. These findings were irrespective of level of global cognitive functioning and expressive and receptive language abilities. After completing the training program, children with SCT show emotion recognition abilities to a level that could not be distinguished from the typically developing group at follow-up.

These findings illustrate that there are opportunities for positively supporting the development of emotion understanding in children with SCT, already at a young age. Given the evidence that in SCT early social cognitive vulnerabilities may emerge and present more profoundly with age (14), early support of early social cognitive development may alter adverse developmental trajectories of young children with SCT, reduce the negative long-term impact of SCT on social adaptive functioning (16, 42).

Improvements in facial emotion recognition were measured with a standardized task which required understanding of facial emotion of real human faces, different from the learned emotions attached on animated vehicles (35). Also, this standardized task gave no information of the emotion in terms of its context, supporting the notion that children with SCT were able to generalize their acquired knowledge during the training program on a distant generalization task. This is especially interesting, as other neurocognitive training programs aimed to enhance emotion recognition in other populations, often show limits in the generalization that were possible to achieve (see for example in ASD populations: (43, 44). The found training effects in the present study were independent of children's global cognitive functioning and their expressive or receptive language skills which are proven to be lower in young children with SCT (45), suggesting that neurocognitive training programs may be suitable and effective for a broad range of young children with SCT.

There were also areas of social cognitive functioning that did not change following the neurocognitive training program, as the study findings indicate that increased emotion recognition abilities after the training did not generalize to direct improvements in social orientation or Theory of Mind. These findings indicate a specific effect of the training on emotion recognition abilities which was the target ability to be trained in the program (27). However, the findings of the current study suggest that The Transporters training program is effective in training understanding of facial emotion in young children with SCT, rather than being effective in enhancing broad early social cognitive development.

Lastly, this study found positive parent and children's perceptions on the feasibility of the training program. The Transporters is an intensive program, expecting the child and parent to invest 45 to 60 min (i.e., three videos accompanied with the exercises from the parent guide) 5 days a week, for a total duration of 4 weeks. Nonetheless, parents were positive about the ability to incorporate the training program in daily life and report that The Transporters was easy to learn and easy accessible. These findings indicate that intensive involvement and guidance of parents during the training program is feasible, which has proven to be effective in generalization and maintenance of learned emotion recognition skills (30). In addition, our findings indicate an intrinsic motivation of children in the SCT training group to watch the animated series, as they reveal that children on average liked the training program from the beginning until the end. These results support the assumption that The Transporters training use intrinsically motivating animated media in a way that children like watching the episodes while learning about emotions in their context (27).

The current study was the first one, to our knowledge, to explore the effectiveness of a neurocognitive training in children with SCT. The inclusion of a training group and two control groups (a SCT waiting list group and a typically developing group), ensured the possibility to check for natural increases or learning effects in social cognitive functioning. Although further research is needed, the current results may contribute to improving clinical care in order to prevent negative long-term impact of SCT on social (cognitive) development. As neurocognitive training programs are easy accessible, cost-effective, and can be used without a clinical indication, support of early neurocognitive development can be preventively executed in home-based and school-based settings. Neurocognitive training programs can also be used as part of an integrative intervention program for young children with SCT at risk of specific social cognitive vulnerabilities, which have become visible based on individual neurocognitive assessment.

While the results of the present study are promising, future research is needed to address its limitations. First, the small sample size of this study especially when it comes to boys with 47, XYY limits the generalizability of the findings. Because of these small samples, we were not able to assess the specific contribution of karyotype (XXX, XXY, XYY) on the efficacy of the training program. Second, the present study only assessed post-training outcome, and did not have a follow-up period to investigate maintenance of the improved abilities and possible longer-term generalization effects. It remains for future studies to evaluate how support of early social cognition is related to functional outcomes, in order to prevent the detrimental impact of the presence of SCT. Replication is therefore necessary in future research, preferable in Randomized Control Trials studies with larger samples sizes and follow-up maintenance assessments, in order to investigate specific effects of neurocognitive training programs within the different karyotypes and longer term training effects. Another promising approach that could be used as a research method complementary to RCTs is the Single Case Experimental Design [SCED; (46)], the appeal of case-based time-series studies, with multiple assessments both before and after intervention. Benefits of SCEDs include the possibility to investigate the efficacy of early intervention in heterogeneous populations (e.g., populations with highly variable phenotypes such as SCT), and being able to test the effectiveness of treatment methods in the complex but real world practice of clinical work.

Taken together, the current study on the efficacy of a neurocognitive training in young children with SCT, an animated facial emotion training program, showed a significant improvement in facial emotion recognition abilities, with a large effect size. Moreover, encouraging results were found with respect to parents' and children's perception on the feasibility of the training program. These findings indicate that it is possible to (preventively) support the development of social cognition in children with an extra X- or Y-chromosome, which may reduce the negative long-term impact of SCT on social adaptive functioning. Additional research is warranted using a larger sample and follow-up maintenance assessments in order to further evaluate the effectiveness of the training for specific subtypes of SCT. Evidence based support of young children with SCT is of great importance given the need for preventive and early training programs, aimed to minimize neurodevelopmental impact.

## Data Availability Statement

The raw data supporting the conclusions of this article will be made available by the authors, without undue reservation.

## Ethics Statement

This study was approved by the Ethical Committee of Leiden University Medical Center, The Netherlands. Signed informed consent was obtained from the parents of all participating children, according to the declaration of Helsinki. Written informed consent to participate in this study was provided by the participants' legal guardian/next of kin. Written informed consent was obtained from the individual(s), and minor(s)' legal guardian/next of kin, for the publication of any potentially identifiable images or data included in this article.

## Author Contributions

NB: design, recruitment of participants, acquisition of data, analysis, interpretation of the data, and drafting. HS: conception, design, and final-approval of the manuscript. SvR: conception, design, interpretation of the data, and final-approval of the manuscript. All authors contributed to the article and approved the submitted version.

## Funding

This work was supported by a grant from the Dutch Organization for Scientific Research (NWO funding # 016.165.397 to SvR).

## Conflict of Interest

The authors declare that the research was conducted in the absence of any commercial or financial relationships that could be construed as a potential conflict of interest.

## Publisher's Note

All claims expressed in this article are solely those of the authors and do not necessarily represent those of their affiliated organizations, or those of the publisher, the editors and the reviewers. Any product that may be evaluated in this article, or claim that may be made by its manufacturer, is not guaranteed or endorsed by the publisher.
