# Supplementary material for: Early Preventive Intervention for Young Children With Sex Chromosome Trisomies (XXX, XXY, XYY): Supporting Social Cognitive Development Using a Neurocognitive Training Program Targeting Facial Emotion Understanding
Source: Front Psychiatry. 2022 Feb 25;13:807793. doi: 10.3389/fpsyt.2022.807793 (PMC8913493; doi:10.3389/fpsyt.2022.807793)
Supplement: Supplementary file 1 [file Table_1.DOCX]

Supplementary Material

*Table 1.* Parents’ response on the 15 items of the Social Validity Questionnaire.

|  | **Negative opinion** | **Neutral opinion** | **Positive opinion** |
| --- | --- | --- | --- |
|  | strongly disagree/disagree | neutral | agree / strongly agree |
| Q1: Learning this intervention model was valuable to my child. | 8.3% | 0% | 91.7% |
| Q2: Using this intervention model was a valuable experience for myself. | 8.3% | 0% | 91.7% |
| Q3: I myself have learned skills from the intervention that help me to stimulate the development of my child. | 8.3% | 0% | 91.7% |
| Q4: My family will continue to use (parts of) this intervention model after finishing the study. | 8.3% | 0% | 91.7% |
| Q5: I would recommend this intervention model to other parents. | 8.3% | 0% | 91.7% |
| Q6: This intervention model was easy to incorporate into my family daily life. | 16.7% | 33.3% | 50% |
| Q7: I have succeeded in applying the homework assignments in daily life (outside of the training). | 16.7% | 16.7% | 66.7% |
| Q8: This intervention model was not complicated to learn. | 0% | 0% | 100% |
| Q9: This intervention model was easy to use. | 16.7% | 0% | 83.3% |
| Q10: I was able to teach other family members to use the strategies I learned, so that they are also able to positively stimulate the development of my child. | 25.0% | 50.0% | 25.0% |
| Q11: This intervention provided significant positive changes for my child. | 8.3% | 8.3% | 83.3% |
| Q12: I noticed meaningful increases in my child’s social interaction with the people in his/her environment. | 16.7% | 41.7% | 41.7% |
| Q13: I noticed meaningful increases in my child’s eye contact with the people in his/her environment. | 16.7% | 75.5% | 8.3% |
| Q14: I noticed meaningful increases in my child’s verbal interaction (babbling or words) with the people in his/her environment. | 16.7% | 83.3% | 0% |
| Q15: Other people noticed a significant positive change in my child. | 41.7% | 50.0% | 8.3% |

*Note. N*=13
